# Supplementary material for: Opioid-related deaths during hospital admissions or shortly after discharge in the United Kingdom: A thematic framework analysis of coroner reports
Source: PLoS One. 2023 Apr 19;18(4):e0283549. doi: 10.1371/journal.pone.0283549 (PMC10115296; doi:10.1371/journal.pone.0283549)
Supplement: Attachment — Submitted filename: response_to_reviews_v1.pdf [file pone.0283549.s001.pdf]

Dr. Dan Lewer
Specialty Registrar in Public Health
Institute of Epidemiology and Healthcare, University College London
1-19 Torrington Place

London WC1E 7HB

d.lewer@ucl.ac.uk

14 February 2023

Re: Opioid-related deaths during hospital admissions or shortly after discharge in the United Kingdom: a thematic framework analysis of coroner reports (PONE-D-22-27646)

Dear Dr Lennox,

Many thanks to you and the reviewer for the feedback and opportunity to revise our article.

We have attached our point-by-point responses, as well as clean and marked-up versions of the revised manuscript.

The authors have declared that no competing interests exist.

Please let us know if you have any questions about this study.

Yours sincerely,

Dan Lewer, Thomas Brothers, Magdalena Harris, Kirsten Rock, and Caroline Copeland

Editor comments

Comment #1. Addressing the balance in the background between objective 1 & 2. Given the issues with the data you may which to consider removing objective 1 and focus on the themes from the reports.

Response: We agree, and we have edited the manuscript and removed objective 1. We feel that the qualitative analysis (objective 2) is the main contribution of this study and the focus of the manuscript. We have retained table 1 as a description of study participants, rather than an analysis of the characteristics of people who died in relevant circumstances (including editing the table title and how it is referenced in the text).

Comment #2. Amend the background to make it clear what is evidenced in research and what are the authors thoughts e.g. this wording 'Potential mechanisms behind the increased risk after hospital discharge have not been studied, but could include' - Also I would recommend removing the reference to newspaper reports and just include academic research evidence.

Response: We have edited and improved the introduction in several ways. This includes: (1) clarifying that there is no existing research into the mechanisms of increased risk of death associated with hospital discharge (to our knowledge); (2) the proposed mechanisms are theories based on research into the hospital risk environment, rather than research into fatal overdoses associated with hospital admission or discharge; (3) removing the references to newspaper reports; (4) a clearer structure; (5) adding references to additional scientific background.

Comment #3. Please include in the background a discussion and rationale for using thematic framework analysis to documents.

Response: We have briefly explained in the introduction that we used a qualitative approach because the coroners' reports include narrative accounts of varying format and length, and provide references to other studies of coroners' reports that have different research questions and use similar methods. In the methods section (under Analysis), we have also extended the section that described thematic framework analysis and why we chose it for this study:

"We used thematic framework analysis to identify and explore factors that may contribute to the risk of fatal overdose around the time of a hospital admission. Framework analysis involves organizing codes into categories developed by the research team; this provides a new structure to the data that is revised and updated as analysis progresses. We chose this method because we had a large number of reports with inconsistent detail; many with just a few words and some with several pages of narrative. The framework analysis method meant that each report could contribute to the framework. We also chose to use framework analysis because wanted to explain a phenomenon (hospital-related overdose risk) rather than develop theory."

Comment #4. Please add to the limitations sections issues around bias.

Response: Thank you for prompting a more thorough discussion of bias. We felt that this question related mainly to selection bias, meaning the representativeness of our sample. This was also raised by the reviewer. The potential sources of selection bias are (a) relevant deaths may not be investigated by a coroner. In the UK, almost all drug-related deaths are investigated, and a death in hospital or shortly after discharge would have an even higher probability of being investigated. We therefore discounted this; (b) some deaths are not submitted to NPSAD. This is a substantial proportion; however participation in NPSAD is at coroner-level rather than individual-level, which is likely to limit bias due to deaths not being submitted; (c) some reports have insufficient information to determine whether a death happened in close proximity to a hospital admission. This is likely to be the main source of selection bias, because coroners may write more detailed reports for certain types of death. They may be more likely to write a detailed account if there are potential questions about the contribution of health services to the death. This may mean that our sample has more deaths involving potential errors/neglect, and fewer where the patient was simply very unwell. Although we cannot be certain of the impact of selection bias, we feel this is likely the main bias in our

study and have edited the discussion to explain this potential limitation. We have removed the text suggesting we do not suspect bias, and we have replaced this with an acknowledgement of the bias and a discussion of its potential impact (please see also comment #7).

Reviewer 1

Comment #5. This study addresses a topic of high importance and relevance, examining the potential mechanisms underlying the increased risk of illicit drug overdose deaths following hospital discharge. Research is needed to help inform strategies for overdose prevention and engagement in harm reduction and other forms of care. However, the novel contribution of this study is unclear, given limitations in the data/approach.

Response: We do feel that these findings are new, and we did this study despite the challenging data because there is very limited research into the causes of fatal overdoses shortly after hospital discharge. The studies referenced in the introduction describe problems with hospital care such as stigma and lack of opioid agonist therapy, rather than analyses of the causes of fatal overdoses during hospital admissions and after discharge (though this led us to hypothesize that the problems with hospital care might contribute to the risk of fatal overdose). In fact, the results of our study are not exactly what we expected. We thought that the most important themes would relate to stigma, delays in opioid agonist provision, opioid withdrawal, and unsafe illicit drug use in hospitals; and therefore the most important interventions would be related to harm reduction, such as improved opioid agonist therapy or hospital-based drug consumption rooms. These themes were evident in our data, but the results suggest that poor physical health is a more important contributor to overdose risk than we expected. In many cases, the decedent was very unwell (for example with cardiac and respiratory illness) and possibly using sedatives to manage symptoms or comorbid mental health problems that were exacerbated by poor physical health. This emphasises the importance of managing chronic health conditions, pain and polysedative use, and palliative care for this group of patients, in addition to harm reduction approaches. This conclusion is highlighted in our abstract and discussion.

Comment #6. The objectives of the study are to describe the characteristics of patients who died due to an opioid overdose during or shortly after discharge, and to identify the contributing factors. With respect to the second objective, the authors note that prior studies tend to rely on hospital data that offer limited contextual information with which to identify factors that contribute to fatal overdoses. However, several potential contributing factors are outlined in the Background/literature review, including some backed with citations of prior research. It isn't clear whether these factors were identified empirically in these prior studies or through speculation, clinical experience, or other means. Further, no attention is given in the Background to establishing a rationale for the first objective. Without this information, the rationale and novel contribution of the current study (which identifies many of these same factors) are not clearly established.

Response: Following your feedback and the editor's feedback (see comment #1), we decided to remove the first objective. We agree with the limitations you discuss in comment #7, and we feel the qualitative analysis is the main contribution of this study. With respect to the second objective, the studies that we reference in the introduction are studies of the hospital risk environment, rather than analyses of factors that might affect the risk of fatal opioid overdose either during a hospital admission or after hospital discharge. The rationale for this study is that we did not know why there is a high risk of opioid overdose at this time; even though factors such as stigma, poor opioid agonist therapy, and zero-tolerance policies have been identified and might contribute. We have edited the introduction to clarify this, and hope it is now clearer.

Comment #7. The data source has limitations that may restrict the impact of this work beyond what is acknowledged in the Discussion. The limited coverage of fatal overdoses, in combination with the voluntary nature of reporting, differences in levels of detail, and unknown (but likely) reporting biases within and across geographical areas and/or coroners, all detract from data quality. Given questions of representativeness, it is not clear that these data are suited to answer the first objective (providing a description of patient characteristics). Some variables in Table 1 have a high level of missing values, hindering interpretation. Further, unknown biases affected what is and isn't recorded across coroners may have impacted the identification of contributing factors. In describing study limitations, the authors note the relatively low coverage of fatal overdoses in the data source but that they do not think there's reason to suspect bias. This seems insufficient justification.

Response: Following your feedback, we have removed objective 1, such that the qualitative analysis is the focus of the study (please see also comment #1). We agree that the potential selection biases limit quantitative inference. We also agree that these selection biases may be important in the interpretation of the qualitative analysis, though there is still substantial valuable data from the coroner reports. We have removed the text suggesting we do not suspect bias, and we have replaced this with an acknowledgement of the bias and a discussion of its potential impact (please see also comment #4).

Comment #8. Use of the risk environments concept fits for this study, but more detail would be appreciated on how this framework was used in the analysis (e.g., what dimensions of context were considered and whether these were limited to hospital contexts). It seems like attention to community contexts may have also been informative, such as what services are available, what is known about the illicit drug supply, its composition and potential variation across communities. Although it makes sense that focus would be placed on hospital contexts, community contexts will also have affected overdoses as well as what gets potential recorded in coroners' reports – was this considered?

Response: We agree that community contexts are also important. The 'hospital risk environment' was one starting point for the deductive aspect of our investigation. When we began coding, we considered the known features of the hospital risk environment (including inadequate treatment of opioid withdrawal and pain, hospital policies that mandate abstinence, structural and interpersonal stigma, poor continuity of care on discharge, and lack of harm reduction supports) and our own expectations of what might cause overdose deaths around the time of a hospital admission. We then analysed the data and generated new themes (ie. the inductive aspect of the research). Our approach therefore did not exclude community contexts, and some of the themes do relate to community contexts (eg. the discharge location, or access to community drug treatment services). We have edited the description of the analysis to clarify the inductive part of the process.

Comment #9. In table 2, the link between the example case and theme isn't always clear; alternative explanations seem possible without more information/context. For example, under 7) poor health leading to reduced access to treatment – is the lack of evidence of recent methadone use (even in the context of poor/worsening health) sufficient to suggest access to treatment was impacted by poor health? This seems speculative, and there are other potential explanations for why OAT was not evident. Perhaps the case description is not fully indicative of the information contained in the report, meaning there were other reasons to attribute this death to a lack of access to treatment. It just isn't clear in what is provided, and so raises questions about how attributions were made during analysis.

Response: Thank you for this advice. We have reviewed the table as a team, and we agree that some of the examples can be improved. We felt that the case summaries for themes 1, 2, 4, 5, 6 and 8 provide a clear and representative example for each theme; however the case summaries for themes 3 and 7 were not clear. We have selected a different case to illustrate theme 3, and modified the case summary for theme 7 to clarify that mobility issues may have affected access to treatment. We agree that there may have been other reasons why OAT was not evident; however the mobility issues clearly appeared relevant, as the patient could not leave their own kitchen unaided.

Journal requirements

Comment #10. Please ensure that your manuscript meets PLOS ONE's style requirements, including those for file naming. The PLOS ONE style templates can be found at https://journals.plos.org/plosone/s/file?id=wjVg/PLOSOne_formatting_sample_main_body.pdf and https://journals.plos.org/plosone/s/file?id=ba62/PLOSOne_formatting_sample_title_authors_affiliations.pdf

Response: We have checked the guidelines and believe that our manuscript meets the style requirements, however please let us know if we need to change anything.

Comment #11. Please provide additional details regarding participant consent. In the ethics statement in the Methods and online submission information, please ensure that you have specified (1) whether consent was informed and (2) what type you obtained (for instance, written or verbal, and if verbal, how it was documented and witnessed). If your study included minors, state whether you obtained consent from parents or guardians. If the need for consent was waived by the ethics committee, please include this information. If you are reporting a retrospective study of medical records or archived samples, please ensure that you have discussed whether all data were fully anonymized before you accessed them and/or whether the IRB or ethics committee waived the requirement for informed consent. If patients provided informed written consent to have data from their medical records used in research, please include this information. Once you have amended this/these statement(s) in the Methods section of the manuscript, please add the same text to the "Ethics Statement" field of the submission form (via "Edit Submission"). For additional information about PLOS ONE ethical requirements for human subjects research, please refer to http://journals.plos.org/plosone/s/submission-guidelines#loc-human-subjects-research.

Response: This is a study of deceased adults. We have supplied PLOS One with a letter from the chair of the King's College London Biomedical & Health Sciences, Dentistry, Medicine and Natural & Mathematical Sciences Research Ethics Sub-Committee confirming that ethics review is not required for this study.

Comment #12. Thank you for stating the following in your Competing Interests section: No. Please complete your Competing Interests on the online submission form to state any Competing Interests. If you have no competing interests, please state "The authors have declared that no competing interests exist.", as detailed online in our guide for authors at http://journals.plos.org/plosone/s/submit-now. This information should be included in your cover letter; we will change the online submission form on your behalf.

Response: Many thanks, we have added this information to our cover letter.

Comment #13. In your Data Availability statement, you have not specified where the minimal data set underlying the results described in your manuscript can be found. PLOS defines a study's minimal data set as the underlying data used to reach the conclusions drawn in the manuscript and any additional data required to replicate the reported study findings in their entirety. All PLOS journals require that the minimal data set be made fully available. For more information about our data policy, please see http://journals.plos.org/plosone/s/data-availability. "Upon re-submitting your revised manuscript, please upload your study's minimal underlying data set as either Supporting Information files or to a stable, public repository and include the relevant URLs, DOIs, or accession numbers within your revised cover letter. For a list of acceptable repositories, please see http://journals.plos.org/plosone/s/data-availability#loc-recommended-repositories. Any potentially identifying patient information must be fully anonymized. Important: If there are ethical or legal restrictions to sharing your data publicly, please explain these restrictions in detail. Please see our guidelines for more information on what we consider unacceptable restrictions to publicly sharing data: http://journals.plos.org/plosone/s/data-availability#loc-unacceptable-data-access-restrictions. Note that it is not acceptable for the authors to be the sole named individuals responsible for ensuring data access. We will update your Data Availability statement to reflect the information you provide in your cover letter.

Response: The raw data for this project are PDF files. Each PDF is a scan of a coroner's report including information from the Record of Inquest, Toxicology Report and Post-Mortem Pathology findings. The NPSAD is a database populated by the information provided in these PDFs in both coded and text fields. Whilst GDPR and the Data Protection Act do not apply to deceased individuals, the details of these reports if made publicly available in their unanonymised form could be distressing for family and close associates of the deceased. NPSAD has therefore specified that these files cannot be made publicly available; however they are available to researchers who agree to certain confidentiality terms. As specified in the manuscript, data access can be arranged by NPSAD by contacting npsad@kcl.ac.uk.

Comment #14. Your ethics statement should only appear in the Methods section of your manuscript. If your ethics statement is written in any section besides the Methods, please delete it from any other section.

Response: The ethics statement only appears in the methods section.
